# Notch and TNF-α signaling promote cytoplasmic accumulation of OLFM4 in intestinal epithelium cells and exhibit a cell protective role in the inflamed mucosa of IBD patients

**DOI:** 10.1016/j.bbrep.2020.100906

**Published:** 2021-01-11

**Authors:** Reiko Kuno, Go Ito, Ami Kawamoto, Yui Hiraguri, Hady Yuki Sugihara, Sayaka Takeoka, Sayaka Nagata, Junichi Takahashi, Mao Tsuchiya, Sho Anzai, Tomohiro Mizutani, Hiromichi Shimizu, Shiro Yui, Shigeru Oshima, Kiichiro Tsuchiya, Mamoru Watanabe, Ryuichi Okamoto

**Affiliations:** a Department of Gastroenterology and Hepatology, Japan; b Advanced Research Institute, Tokyo Medical and Dental University (TMDU), Tokyo, Japan; c Center for Stem Cell and Regenerative Medicine, Tokyo Medical and Dental University (TMDU), Tokyo, Japan

**Keywords:** Tumour necrosis factor-α (TNF-α), Notch pathway, OLFM4, IBD, inflammatory bowel disease, UC, ulcerative colitis, CD, Crohn's disease, Dox, doxycycline, NICD, Notch intracellular domain, qRT-PCR, quantitative reverse transcription-polymerase chain reaction analysis, TNF-α, tumour necrosis factor α, DBZ, intestinal epithelial cells, IEC, dibenzazepine, ChIP, chromatin immunoprecipitation

## Abstract

Notch signaling is activated in the intestinal epithelial cells (IECs) of patients with inflammatory bowel disease (IBD), and contributes to mucosal regeneration. Our previous study indicated that TNF-α and Notch signaling may synergistically promote the expression of the intestinal stem cell (ISC) marker OLFM4 in human IECs. In the present study, we investigated the gene regulation and function of OLFM4 in human IEC lines. We confirmed that TNF-α and Notch synergistically upregulate the mRNA expression of OLFM4. Luciferase reporter assay showed that OLFM4 transcription is regulated by the synergy of TNF-α and Notch. At the protein level, synergy between TNF-α and Notch promoted cytoplasmic accumulation of OLFM4, which has potential anti-apoptotic properties in human IECs. Analysis of patient-derived tissues and organoids consistently showed cytoplasmic accumulation of OLFM4 in response to NF-κB and Notch activation. Cytoplasmic accumulation of OLFM4 in human IECs is tightly regulated by Notch and TNF-α in synergy. Such cytoplasmic accumulation of OLFM4 may have a cell-protective role in the inflamed mucosa of patients with IBD.

## Highlights

• Notch and TNF-α signaling is important in IECs of patients with IBD.
• Notch and TNF-α signaling promotes the cytoplasmic accumulation of OLFM4.
• OLFM4 accumulation may have anti-apoptotic properties.
• OLFM4 could protect against mucosal inflammation in IBD.

## 1 Introduction

Inflammatory bowel disease (IBD) is a chronic inflammatory disorder that can be further categorized into ulcerative colitis (UC) and Crohn's disease (CD). These diseases develop from multiple factors, including genetic susceptibility, immune dysfunction, and dysbiosis [1]. The intestinal epithelial cells (IECs) are also involved in the pathogenesis of IBD [2].

The intestinal epithelium is a monolayer tissue consisting of crypts and villi. Genuine intestinal stem cells (ISCs) reside at the bottom of crypts. These ISCs can differentiate and give rise to absorptive or secretory progenitor cells, depending on Notch activity [3]. Thus, Notch signaling is essential for the maintenance of the intestinal epithelium; however, it remains unclear how it influences the pathogenesis of IBD.

Notch signaling is activated by the binding of a Notch ligand to the receptor between neighboring cells, which results in the release of the Notch intracellular domain (NICD) by γ-secretase-dependent proteolysis [4]. Subsequently, NICD translocates into the nucleus, forms a complex with RBP-J, and binds to target DNA sites to promote the transcription of target genes, such as Hes1. We previously reported that Notch signaling is activated in the inflamed mucosa of IBD patients, and contributes to regeneration and proliferation [5]. In addition, we found that TNF-α reciprocally enhances cell-intrinsic Notch activity in human IECs [6]. A comprehensive analysis identified a group of genes synergistically regulated by TNF-α and Notch signaling, including olfactomedin-4 (OLFM4) [6], which encode a 72-kDa glycoprotein belonging to the olfactomedin family [7]. OLFM4 is a robust stem cell marker for human IECs [8], and is a direct target of Notch signaling in IECs [9]. However, in myeloid cells, OLFM4 expression is regulated by activation of the NF-κB pathway [10]. OLFM4 expression is upregulated in the inflamed mucosa of IBD patients [11,12]; however, the precise regulatory mechanisms and its functional role in IBD remain unclear.

Recent advances in organoid culture have enabled the functional analysis of untransformed human IECs [13]. We therefore analyzed the regulation of the OLFM4 gene using human IEC lines and patient-derived organoids. In addition, the functional role of cytoplasmic OLFM4 was examined in the context of IBD.

In the present study, we aimed to elucidate the precise regulatory mechanism of OLFM4 gene expression in human IECs under the inflammatory environment. Also, our hypothesis on the functional role of OLFM4 accumulation in the cytoplasm of human IECs was tested by analyses using human IEC cell-lines, patient-derived organoids, and patient-derived tissues.

## 2 Materials and methods

### 2.1 Human intestinal tissue specimens

Human tissue specimens were obtained from patients who underwent endoscopic examination or surgery at Yokohama Municipal Citizen's Hospital or Tokyo Medical and Dental University Hospital from 2012-2020. Written informed consent was obtained from each patient, and the study was approved by the ethics committee of Yokohama Municipal Citizen's Hospital and Tokyo Medical and Dental University Hospital. Normal intestinal tissue as the control was obtained from a non-inflamed region of the small intestine of patients with Crohn's disease or UC.

### 2.2 Cell culture

Cell lines expressing the Notch1 intracellular domain (tet-on NICD cells) under the control of doxycycline (DOX, 100 ng/mL, Clontech) were generated from LS174T cells and DLD1 cells as described previously [14]. Establishment of patient-derived intestinal organoids was performed as described previously [6]. Reagents or pro-inflammatory factors used for organoid culture-based experiments are listed in Supplementary Tables S1 and S2. Cell Recovery Solution (Corning) was used to harvest the organoids. All the organoids underwent 4 passages prior to analysis.

### 2.3 Immunohistochemistry

Immunohistochemistry using intestinal tissues was performed according to a previously published method [15]. Briefly, frozen sections were prefixed with 4% PFA, and cryosections of 8 μm thickness were obtained. Antigen retrieval was performed using microwave treatment (500 W, 10 min) in 10 mM citrate buffer for immunofluorescent detection of OLFM4, NF-κB p65, and NICD1. Primary antibodies are listed in Supplementary Table S3. Tyramide-based signal amplification was used for the detection of each antigen (Molecular Probes). Tissues were counterstained with 4′, 6-diamidino-2- phenylindole (DAPI). Data were collected using an epifluorescent microscope (BZ-X700, KEYENCE) or a confocal microscope (FLUOVIEW FV10i, OLYMPUS).

### 2.4 Quantitative RT-PCR

RNA extraction from cultured cells or organoids, reverse transcription, and SYBR-green based qPCR were performed as described previously [6]. The primer sequences for human β-actin and OLFM4 have also been previously described [6,15]. Expression levels were normalized to those of β-actin, and fold-change was calculated relative to the control.

### 2.5 Western blot analysis

Cell lysis and protein extraction were performed in SDS-RIPA buffer described previously [6]. Primary antibodies are listed in Supplementary Table S3. Probed blots were visualized with Luminata Forte western HRP substrate (Millipore) and images were captured using the ChemiDoc imaging system (Bio-Rad Laboratories).

### 2.6 ELISA

Cells were seeded at 1 × 10^5^ cells/well on a 12-well plate. After 24 h, cells were stimulated with DOX (100 ng/mL, Clontech) for 48 h and/or recombinant human TNF-α (50 ng/ml, PeproTech) for 24 h. Supernatants were collected at the end of the stimulation period. OLFM4 levels were determined using an enzyme-linked immunosorbent assay (ELISA) kit for olfactomedin 4 (Cloud-Clone Corp).

### 2.7 Luciferase-based reporter assays

For all assays, cells were seeded onto 12-well plates at 1 × 10^5^ cells/well 24 h prior to transfection. Luciferase-based reporter assays were performed as described previously [16]. For determining the transcriptional level of OLFM4, cells were co-transfected with 1.5 μg of OLFM4-Luc vector using Trans-IT LT1 reagent (Mirus Bioresearch). After 24 h of incubation with Dox and/or TNF-α (50 ng/mL), IL-1β (25 ng/mL), and IFN-γ (50 ng/ml), cells were analyzed using the Dual-Luciferase Reporter Assay System (Promega) according to the manufacturer's instructions. Luminescence was measured using a GloMax Discover Microplate Reader (Promega). All experiments were carried out in triplicate, and the results are shown as the mean ratio of firefly and renilla luciferase activity.

### 2.8 Chromatin immunoprecipitation analysis

Cells were stimulated using DOX (100 ng/mL) and/or recombinant human TNF-α (50 ng/mL) for 24 h. Chromatin immunoprecipitation (ChIP) was performed using the Simple ChIP enzymatic Chromatin IP kit (on magnetic beads, Cell Signaling Technology) according to the manufacturer's instructions. For each immunoprecipitation reaction, 10 μg of chromatin was used. Antibodies for immunoprecipitation were as follows: anti-human cleaved Notch1 (1:200, Cell Signaling Technology), anti-human p65 (1:100, Cell Signaling Technology), or normal rabbit IgG (2μg) as the negative control. Primer sequences used for DNA amplification are indicated in Supplementary Fig. S5. qPCR analysis of the precipitated DNA was performed in triplicate.

### 2.9 Plasmids

Construction of a luciferase reporter plasmid (OLFM4-Luc) harboring a 2 kb sequence of the human OLFM4 gene promoter region (-2000 to +10) was constructed using a PCR-based method as described previously [16]. Corresponding DNA fragments were amplified from human genomic DNA by PCR and inserted into the pGL4.10[luc2] vector (Promega), between the KpnI and NheI sites. The nucleotide position of the translation start site was defined as the +1 position. The expression vector for OLFM4 was purchased from ORIGENE (RC214942).

### 2.10 Data presentation

Unless otherwise indicated, quantitative data are displayed as mean ± SEM from at least three independent experiments. Significance was assessed using an unpaired Student's t-test and a cut-off P-value < 0.05.

## 3 Results

### 3.1 OLFM4 protein expression is regulated by TNF-α and notch activation in human IECs

**Fig. 1:**
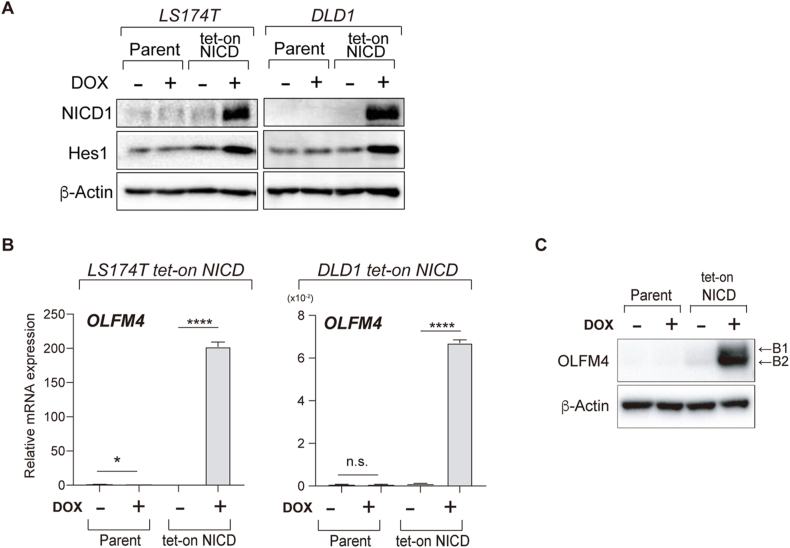
OLFM4 expression is enhanced by Notch activation. Cells were stimulated with dH_2_O (control) or doxycycline (DOX, 100 ng/ml) for 24 h unless otherwise stated. (A) LS174T and DLD1 parent cells (Parent), and their respective tet-on NICD cells (NICD) were treated with DOX and collected for immunoblot analysis of NICD1 and Hes1. (B) Cells were treated with DOX and collected for qRT-PCR analysis of OLFM4 expression. data were normalized to β-actin levels. **P < 0.01; ****P < 0.0001. n.s. not significant. (C) LS174T tet-on NICD cells were treated with DOX and collected for immunoblot analysis of intracellular OLFM4 protein. Two different forms of OLFM4 protein (B1 and B2) were observed.

In our previous studies, we established human IEC lines in which Notch activation could be induced by the addition of DOX (tet-on NICD cells) [6,14]. Such cells were generated from two human colonic IEC lines, LS174T (LS174T tet-on NICD cells) and DLD1 (DLD1 tet-on NICD cells). Immunoblot analysis of these cells confirmed a clear induction of NICD1 expression upon DOX addition, and up-regulation of Hes1 (Fig. 1A). Using these cell lines, we measured the expression levels of the ISC marker gene, OLFM4 (Fig. 1B). OLFM4 mRNA expression was significantly upregulated (LS174T tet-on NICD cells, 200 fold, P < 0.0001; DLD1 tet-on NICD cells, 7 fold, P < 0.0001) by Notch activation. Immunoblot analysis also confirmed that intracellular OLFM4 protein levels were significantly upregulated by the induction of Notch activation in LS174T tet-on NICD cells (Fig. 1C). Consistent with other cell types, OLFM4 protein existed in at least two distinct forms in human IECs. To identify the differences between these forms, cell lysates were exposed to PNGaseF digestion (Supplementary Fig. S1). A clear shift was observed from the high molecular weight OLFM4 (B1) to the low molecular weight OLFM4 (B2), confirming that the OLFM4 protein can exist in both N-glycosylated and de-glycosylated forms in human IECs.

**Fig. 2:**
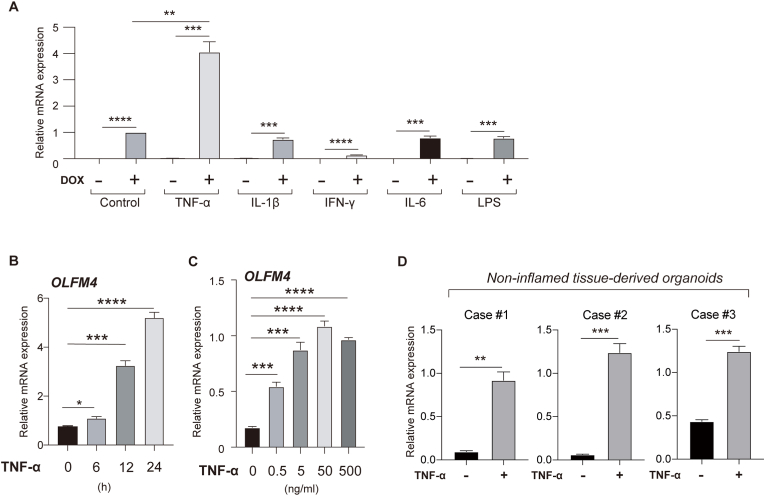
OLFM4 expression is enhanced by the synergy of TNF-α and Notch activation. (A) Cells were treated with DOX, TNF-α (50 ng/ml), IL-1β (25 ng/ml), IFN-γ (50 ng/ml), IL-6 (50 ng/ml), or LPS (100 ng/ml) for 24 h and collected for qRT-PCR analysis of OLFM4 expression. (B) After pre-treatment with DOX (100 ng/ml) for 24 h, LS174T cells were treated with TNF-α (50 ng/ml) for the indicated time-period. (C) After pre-treatment by DOX (100 ng/ml) for 24 h, LS174T cells were treated with TNF-α at the indicated concentration for 24 h. (D) Colonic organoids established from non-inflamed human colonic tissue (3 cases) were treated with TNF-α (50 ng/ml) for 24 h and collected for qRT-PCR analysis of OLFM4 expression. Data were normalized to β-actin levels. *P < 0.05; **P < 0.01; ***P < 0.001; ****P < 0.0001.

In the inflamed mucosa of IBD patients, IECs are exposed to various pro-inflammatory cytokines. Thus, we further examined whether any of these pro-inflammatory cytokines can modulate the expression of OLFM4 in human IECs. A total of 5 pro-inflammatory cytokines/stimuli were tested (Fig. 2A). Among them, TNF-α showed a clear synergistic effect with Notch activation (DOX^+^ vs DOX^+^TNF-α^+^, 4.06 fold, P = 0.0014) on the expression of OLFM4. The TNF-α-induced activation of the NF-κB pathway in LS174T tet-on NICD cells was confirmed (Supplementary Fig. S2). The effect of TNF-α was confirmed in the parent LS174T cells (Supplementary Fig. S3), and the effect of TNF-α was time- (Fig. 2B) and dose-dependent (Fig. 2C).

To further confirm the effect of TNF-α/Notch synergy on OLFM4 expression in human IECs, we employed a human colonic organoid system. Under standard culture conditions, cell-intrinsic Notch activity is maintained at a high level in these human colonic organoids [6]. Consistently, a clear up-regulation of OLFM4 expression (Case1, 9.6 fold, P = 0.0011; Case2, 20.5 fold, P = 0.0003; Case3, 2.85 fold, P = 0.0002) following the addition of TNF-α was confirmed in patient-derived colonic organoids (Fig. 2D). Inhibition of cell-intrinsic Notch activity also clearly led to a reduction in the intracellular protein levels of OLFM4 (Supplementary Fig. S4).

These results collectively indicate that OLFM4 expression is regulated by TNF-α/Notch synergy in human IECs.

### 3.2 OLFM4 gene transcription is regulated by TNF-α/notch synergy in human IECs

**Fig. 3:**
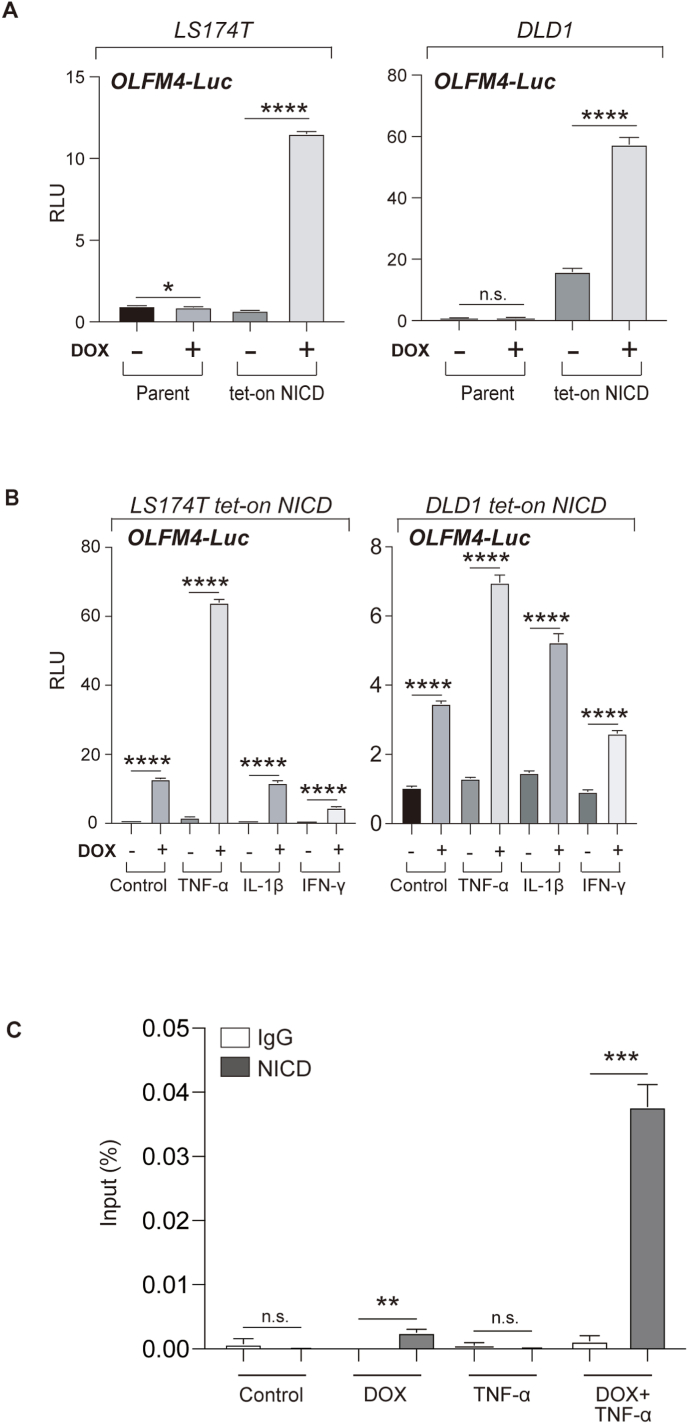
Increased expression of OLFM4 is regulated at the transcriptional level by TNF-α and Notch activation in human IECs.Cells were stimulated with dH_2_O (control) or DOX (100 ng/mL) for 24 h, unless otherwise indicated. (A) Luciferase reporter analysis using OLFM4-Luc. The transcriptional activity of the human *OLFM4* gene was quantified in LS174T tet-on NICD and DLD1 tet-on NICD cells using a luciferase reporter plasmid containing the -2000 to +10 region of the human *OLFM4* gene. (B) Luciferase reporter analysis using OLFM4-Luc with the addition of cytokines to LS174T tet-on NICD cells. (C) A ChIP assay for the human OLFM4 promoter region was performed in LS174T tet-on NICD cells. Cells were stimulated with DOX and TNF-α (50 ng/mL) for 24 h and subjected to ChIP analysis. Immunoprecipitation was performed using either rabbit IgG or anti-NICD1 antibodies. Primer sets were designed to amplify the proximal region of the human OLFM4 promoter, including an area with putative binding sites for RBP-Jκ and NF-κB (Site A). Data were normalized to the initial chromatin input. *P < 0.05; **P < 0.01; ***P < 0.001; ****P < 0.0001, n.s. not significant.

We measured *OLFM4* transcription to clarify the mechanism of the synergistic regulation by TNF-α and Notch. To measure the transcriptional activity of the *OLFM4* gene, we constructed a luciferase-based reporter harboring 2000 bp (-2000 to +10) of the *OLFM4* gene promoter region (OLFM4-Luc). Using this reporter, we performed *OLFM4* gene reporter assays in human IEC lines. Upon the addition of DOX, the activity of OLFM4-Luc was significantly upregulated in LS174T tet-on NICD cells (16.4 fold, P < 0.0001) and in DLD1 tet-on NICD cells (3.6 fold, P < 0.0001) (Fig. 3A), confirming that *OLFM4* is directly regulated by NICD [9].

Next, we examined whether TNF-α/Νοtch synergy also regulates the *OLFM4* gene at the transcription level. OLFM4-Luc assays confirmed significant promotion of OLFM4 transcription activity in LS174T tet-on NICD cells (34.6 fold, P < 0.0001) and in DLD1 tet-on NICD cells (5.3 fold, P < 0.0001) by TNF-α/Νοtch synergy (Fig. 3B). Previous studies have identified key promoter sequences in the proximal region (-101 to +10) of the *OLFM4* gene (Supplementary Fig. S5). In the subject region (Site A), at least 1 putative RBP-Jκ binding site and 1 putative NF-κB binding site were identified [9,10]. Thus, we performed a ChIP of this promoter region, focused on the binding of NICD and NF-κB (p65). We observed binding of NICD within the site A region following Notch activation alone (Fig. 3C). In addition, binding of NICD was significantly enhanced (DOX^+^TNF-a^+^, IgG vs NICD, 29.5 fold, P = 0.0005) by TNF-α/Νοtch synergy. In contrast, NF-κB binding to the site A region was not clearly detected by TNF-α alone or TNF-α/Νοtch synergy (Supplementary Fig. S6). These results suggest that enhanced binding of NICD at the proximal region of the human OLFM4 gene may be one of the key events in TNF-α/Νοtch synergy-mediated promotion of OLFM4 expression. However, it remains uncertain whether a molecular interaction between Notch and NF-κB is required for the functional outcome of TNF-α/Notch synergy in human IECs.

### 3.3 TNF-α/Notch synergy promotes cytoplasmic accumulation of OLFM protein in human IECs

**Fig. 4:**
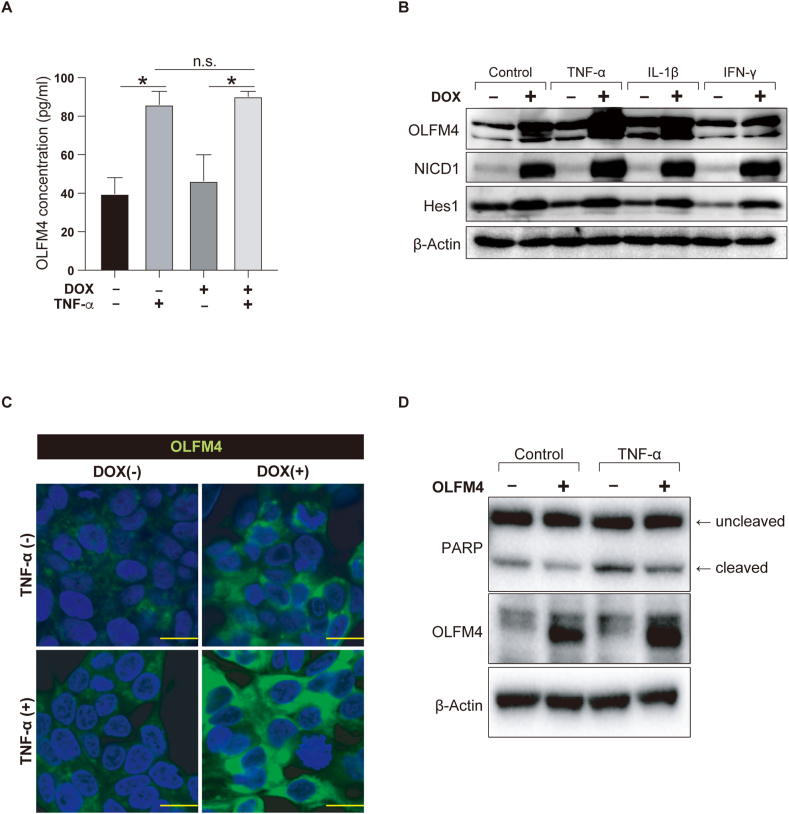
Synergy between TNF-α and Notch activation promotes cytoplasmic accumulation of OLFM4 protein in human IECs. (A) LS174T tet-on NICD cells were treated with DOX and TNF-α (50 ng/ml) for 24 h, before the supernatants were collected for ELISA. The secretion levels of the OLFM4 protein are indicated. (B) LS174T tet-on NICD Cells were treated with DOX and TNF-α (50 ng/ml), IL-1β (25 ng/ml), or IFN-γ (50 ng/ml) for 24 h before immunoblot analysis. Protein levels of OLFM4, NICD1, and Hes1 are shown. (C) LS174T tet-on NICD Cells were treated with DOX and TNF-α (50 ng/ml) for 24 h before immunostaining for OLFM4 (green). Scale bar, 10 μm. (D) Apoptotic response under transient overexpression of cytoplasmic OLFM4 in LS174T cells. Cells were treated with TNF-α (50 ng/ml) for 24 h before collection for immunoblot analysis. Levels of PARP and OLFM4 are shown. *P < 0.05; n.s. not significant. (For interpretation of the references to colour in this figure legend, the reader is referred to the Web version of this article.)

TNF-α/Notch synergy strongly promotes OLFM4 expression at the transcriptional level; however, its functional role in the inflammatory environment was unclear. The OLFM4 protein can be secreted or localized to the cytoplasm [17]. Thus, we first examined whether TNF-α/Notch synergy can increase OLFM4 protein secretion. OLFM4 protein secretion in LS174T tet-on NICD cells increased significantly (DOX^-^TNF-α^-^ vs DOX^-^TNF-α^+^, 2.17 fold, P = 0.0339) after the addition of TNF-α (Fig. 4A). However, activation of Notch did not induce an additional increase in OLFM4 protein secretion under co-stimulation with TNF-α.

In contrast, immunoblot analysis of LS174T tet-on NICD cells showed an increase in the intracellular OLFM4 protein levels via TNF-α/Notch synergy (Fig. 4B). Immunostaining of LS174T tet-on NICD cells showed a consistent pattern, confirming cytoplasmic accumulation of the OLFM4 protein via TNF-α/Notch synergy (Fig. 4C).

Secretory-type OLFM4 may exhibit antibacterial properties [12], while cytoplasmic OLFM4 may exhibit anti-apoptotic activity [17]. To identify the functional role of cytoplasmic OLFM4 accumulation, we analyzed LS174T cells in which transient overexpression of cytoplasmic OLFM4 was induced (Fig. 4D). Immunoblot analysis showed a reduction in cleaved PARP levels (Control, OLFM4^-^ vs OLFM4^+^, 1057147.2 vs 794867.2 based on densitometry) following the overexpression of cytoplasmic OLFM4. Suppression of PARP cleavage was also observed under TNF-α stimulation (TNF-α, OLFM4^-^ vs OLFM4^+^, 1799036.0 vs 1310414.4 based on densitometry), indicating the anti-apoptotic role of cytoplasmic OLFM4 in human IECs in inflammatory environments.

In summary, our results revealed that TNF-α promotes OLFM4 secretion by human IECs. In contrast, TNF-α/Notch synergy can promote the accumulation of cytoplasmic OLFM4, and thus exert anti-apoptotic properties with a cell-protective role in the inflammatory environment. These results suggest that OLFM4 has a distinct role from other stem cell markers, and plays an important role in maintaining epithelial homeostasis in the inflammatory environment.

### 3.4 Cytoplasmic accumulation of OLFM4 is induced in the inflamed mucosa of IBD patients

To confirm the clinical relevance of our *in vitro* results, we examined whether cytoplasmic accumulation of the OLFM4 protein could be observed in patient-derived organoids or in patient-derived tissue under inflammatory conditions.

**Fig. 5:**
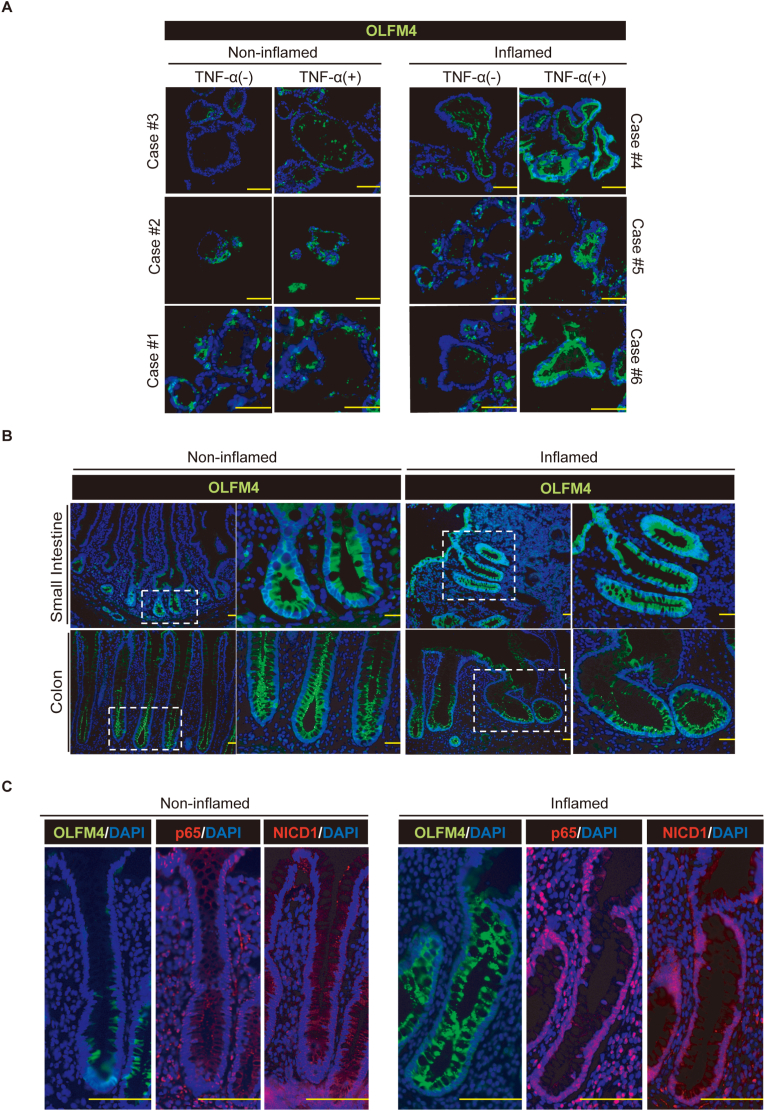
OLFM4 expression is enhanced and accumulates intracellularly in IECs of IBD patients. (A) Colonic organoids from patients were treated with TNF-α (50 ng/ml) for 24 h, before immunostaining for OLFM4 (green). (B) Inflamed and non-inflamed tissues from the small intestine and colon of patients were immunostained for OLFM4 expression (green). Small intestinal tissue of a patient with Crohn's disease and colon tissue of a patient with ulcerative colitis (UC) were used to show representative inflammatory patterns of OLFM4 expression. An enlarged view of an area of the left-side panel (white dotted square) is shown in the right panel. (C) Non-inflamed and inflamed colon tissues of a patient with UC were stained for OLFM4 (green), p65 (red), and NICD1 (red) using serial sections. Scale bar, 100 μm. All tissues or organoids were counterstained using DAPI (blue). (For interpretation of the references to colour in this figure legend, the reader is referred to the Web version of this article.)

We compared the response to TNF-α in a series of patient-derived organoids (Fig. 5A). Organoids established from non-inflamed mucosa showed minimal accumulation of cytoplasmic OLFM4 protein in response to TNF-α. However, in sharp contrast, organoids derived from the inflamed mucosa of patients with IBD showed clear cytoplasmic accumulation of the OLFM4 protein in response to TNF-α.

In patient-derived tissue, the expression of OLFM4 was strictly limited to the crypt-base stem cell area in non-inflamed intestinal tissue (Fig. 5B). However, in the inflamed mucosa of patients with IBD, OLFM4 was expressed throughout the crypt-villi axis with cytoplasmic accumulation in each IEC. The up-regulation and cytoplasmic accumulation of the OLFM4 protein was observed preferentially in the IECs, where nuclear localization of p65 and NICD1 was observed (Fig. 5C). These results suggest that the TNF-α/Notch synergy may play an essential role in cytoplasmic accumulation of OLFM4 in IECs in the inflamed mucosa of patients with IBD, which may have a cell-protective function.

## 4 Discussion

In the present study, we highlighted the importance of TNF-α/Notch synergy in the transcriptional regulation of OLFM4 in human IECs. We observed a significant increase in NICD binding to the corresponding promoter region, suggesting that an unknown factor may recruit NICD under a TNF-α/NF-κB-pathway active context.

The product of the *OLFM4* gene is a glycoprotein secreted by IECs and may act as an anti-microbial peptide by binding to defensins [12]. It is previously established that a common N-glycosylation is added post-translationally to OLFM4 in myeloid cells [10]. We newly confirmed this in human IECs in the present study (Fig. 1C and Supplementary Fig. S1).

Secreted OLFM4 may exhibit anti-microbial activity by binding to defensins [12]. We additionally found that cytoplasmic OLFM4 might also have an anti-apoptotic role in untransformed human IECs. OLFM4-deficient mice did not show any spontaneous colonic phenotypes [18]. However, increased carcinogenesis was observed in OLFM4-deficiency under the APC^min^ background [19]. In human patients, the expression level of OLFM4 is upregulated in early stage colon cancer, but is reduced or lost in advanced-colon cancer [20]. These results suggest that OLFM4 may have a greater importance in pathogenic contexts, such as inflammation and tumorigenesis.

In IECs, OLFM4 expression may be induced by activation of the Wnt pathway [19]. As most of our data were obtained in a Wnt-high context, a detailed analysis of the role of Wnt, Notch, and the TNF-α pathway may reveal how OLFM4 expression is regulated by these key pathways.

In addition to previous studies, our present study has featured the importance of TNF-α/Notch synergy in the transcriptional regulation of OLFM4. We showed that the proximal promoter region harboring the putative NF-κB binding site is essential for the transcriptional regulation by TNF-α/Notch synergy. However, our ChIP assay failed to demonstrate a clear increase of NF-κB (p65) binding to the corresponding promoter region (Supplementary Fig. S6). Instead, a significant increase in NICD binding to the corresponding promotor region was observed, indicating an unknown factor may recruit NICD under an NF-κB-active context.

Among various inflammatory stimuli, IFN-γ seemed to downregulate OLFM4 expression (Fig. 2C). A previous report identified IFN-γ as one of the main mediators of Paneth cell degranulation or induction of apoptosis [21]. Paneth cell loss can also be induced by IFN-λ [22]. Loss of Paneth cells via IFNs may lead to the downregulation of Notch signal-sending cells, resulting in down-regulation of OLFM4 expression in intestinal stem cells.

In conclusion, we showed that TNF-α/Notch synergy leads to enhanced OLFM4 transcription in human IECs. In the inflamed mucosa of IBD patients, TNF-α/Notch synergy may promote cytoplasmic accumulation of OLFM4 protein, which can exhibit anti-apoptotic properties to protect IECs under inflammatory environments.

## Authorship Statement

**Reiko Kuno:** Investigation, Data curation, Writing- Original draft preparation. **Go Ito:** Conceptualization, Investigation, Methodology**. Ami Kawamoto:** Investigation, Data curation, Writing- Original draft preparation. **Yui Hiraguri:** Investigation, Data curation**, Hady Yuki Sugihara:** Investigation, Data curation**, Sayaka Takeoka:** Investigation, Data curation**, Sayaka Nagata:** Investigation, Data curation, Validation **, Junichi Takahashi:** Data curation, Validation **, Mao Tsuchiya:** **Data curation, Validation, Sho Anzai:** **Data curation, Validation, Tomohiro Mizutani:** Methodology, Validation, Visualization**, Hiromichi Shimizu:** Methodology, Validation**, Shiro Yui:**Methodology, Validation**, Shigeru Oshima:** Methodology, Validation**, Kiichiro Tsuchiya:** Methodology, Validation**, Mamoru Watanabe:** Supervision**, Ryuichi Okamoto:** Writing- Reviewing and Editing, Conceptualization.

## Declaration of competing interest

Authors have no conflict of interest to declare.
