# Supplementary material for: Notch and TNF-α signaling promote cytoplasmic accumulation of OLFM4 in intestinal epithelium cells and exhibit a cell protective role in the inflamed mucosa of IBD patients
Source: Biochem Biophys Rep. 2021 Jan 11;25:100906. doi: 10.1016/j.bbrep.2020.100906 (PMC7808948; doi:10.1016/j.bbrep.2020.100906)
Supplement: Multimedia component 1 [file mmc1.pdf]

Supplementary Table S1 List of factors and matrix used to culture organoids.

Material	Company	Product code	Final concentration
Advanced-DMEM	Thermo Fisher	2028-EG	50%
L-WRN-Conditioned medium	ATCC	CRL-3276™	50%
Recombinant human EGF	PeproTech	315-09	50 ng/ml
A83-01	Tocris	2939	500 µM
Y-27632	Wako	030-24021	10 µM
Recombinant IGF-1	Biolegend	590906	100 ng/ml
Recombinant FGF	PeproTech	100-18B	50 ng/ml
Matrigel	Corning	356231	-

Supplementary Table S2 List of pro-inflammatory factors used in the present study.

Material	Company	Product code	Final concentration
recombinant human TNF-α	PeproTech	300-01A	50 ng/ml
recombinant human IFN-γ	PeproTech	300-02	50 ng/ml
recombinant human IL-1β	R&D Systems	201-LB	25 ng/ml
recombinant human IL-6	R&D Systems	206-IL	50 ng/ml
lipopolysaccharides from *E. Coli*	Sigma-Aldrich	L4005	100 ng/ml

Supplementary Table S3 Primary antibodies used in the present study

Gene	Company	Product code	Analysis	Final Concentration
anti-human OFM4	Cell Signaling Technology	D1E4M	Immnnostaining	1:1000
anti-phospho-NF-κBp65	Cell Signaling Technology	3037	Immnnostaining	1:50
anti-cleaved Notch1	Cell Signaling Technology	4147S	Immnnostaining	1:1000
anti-human OLFM4	IMGENEX	IMG-5983A	Immunoblot	1:500
anti-human OLFM4	Cell Signaling Technology	D1E4M	Immunoblot	1:1000
anti-cleaved Notch1	Cell Signaling Technology	4147S	Immunoblot	1:1000
anti-Hes1	Toray (kind gift from Dr. Tetsuo Sudo*)	-	Immunoblot	1:3000
anti-β-actin	Sigma-Aldrich	A5441	Immunoblot	1:16000
anti-IκB	Cell Signaling Technology	9242	Immunoblot	1:1000
anti-phospho-NF-κBp65	Cell Signaling Technology	3037	Immunoblot	1:1000
anti-human PARP	Cell Signaling Technology	9542	Immunoblot	1:1000

* Ito T, Udaka N, Yazawa T, Okudela K, Hayashi H, Sudo T, Guillemot F, Kageyama R, Kitamura H. Basic helix-loop-helix transcription factors regulate the neuroendocrine differentiation of fetal mouse pulmonary epithelium. Development. 2000 Sep;127(18):3913-21.

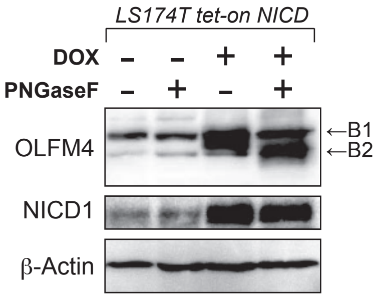

Supplementary Fig. S1 OLFM4 protein expression in LS174T cells.
Cell lysates of Fig. 1C were treated with PNGaseF (230 U/mL) for overnight at 37 °C. Immunoblot analysis for OLFM4, NICD1 and β-Actin are shown. High molecular-weight form (B1) and low molecular-weight form (B2) are identified for the OLFM4 protein.

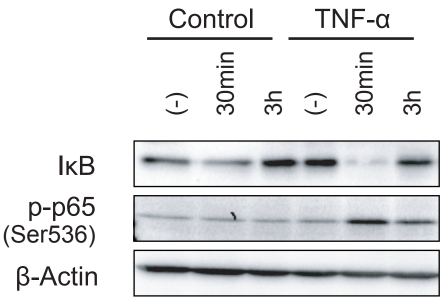

Supplementary Fig. S2. Activation of the NF-κB pathway by TNF-α in LS174T cells. LS174T tet-on NICD cells were treated with TNF-α (50 ng/ml)for the indicated time period, and collected for immunoblot analysis of IκB, phospho-p65, and β-Actin.

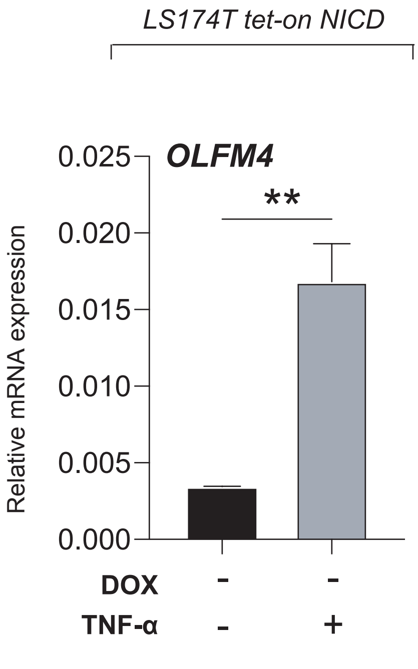

Supplementary Fig. S3. TNF-α up-regulates OLFM4 mRNA expression in LS174T cells. LS174T cells were treated with TNF-α (50 ng/ml) for 24 h and collected for qRT-PCR analysis of OLFM4 expression. Data were normalized to the expression level of β-actin. **P < 0.01.

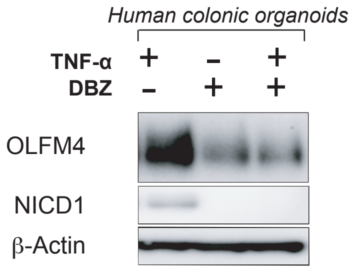

Supplementary Fig. S4 OLFM4 protein expression in human organoids.
Human colonic organoids derived from non-IBD (Normal) patients were cultured in standard media. Organoids were pre-treated for 48 h with a γ-secretase inhibitor,dibenzazepine (DBZ, 10 μM), for Notch inhibition in a medium depleted with Wnt3a, R-Spondin1, nicotinamide, A83-01, and SB202190 (EN+DBZ). Organoids were then treated with TNF-α (50 ng/ml) for 24 h. Immunoblot analysis for OLFM4, NICD1 and β-Actin is shown.

Putative NF-κB binding site (Ref.10)
Putative RBP-Jκ binding site (Ref. 9)

-300 TGTCATCCACAAAGACTACTTTGGTCTCATCTCTTTCAAGGAAGTACCAAGTGGGAAACCAAATATTGGGCTTTACAAAGCACATTTGAGTTAATGTTTG

Site A

-200 GCAGGGGATATCTGGATCCAAGGGCAGCTCACAGAGTGGGCCTCACTGACAAGGTGTGGGCCATGACTCAGATTCCTGGGTGTCCTGAACCTCCTGGGGC

Forward primer

-100 AGTTCACACGTTCCCTGGCCACTCCCTCTTTTCCTACATGCTGGCCATGGGGAAATCACCACTGGGCACTATAAGAAGCCCCTGGGCTCTCTGCAGAGCC

Transcription Start Site Translation Start Site

+1 AGCGGCTCCAGCTAAGAGGACAAG**ATG**AGGCCCGGCCTCTCATTTCTCCTAGCCCTTCTGTTCTTCCTTGGCCAAGCTGCAGGGGATTTGGGGGATGTGG

Reverse primer

Supplementary Fig. S5. Proximal promotor region analyzed in the ChIP assay.
Nucleic acid sequence for the promoter region and the 5' -UTR region of human OLFM4 gene is shown. Putative binding sites of RBP-Jκ (purple) and NF-κB (blue), as well as details of sites that where amplified by primers designed for ChIP analysis (Site A, underline)are indicated.

Kuno *et al*
Fig. S5

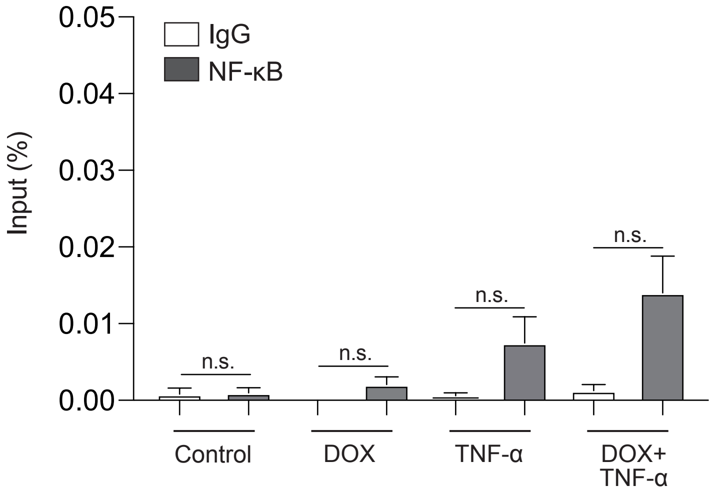

Supplementary Fig. S6. ChIP assay for the proximal promotor region of OLFM4 gene. A ChIP assay for the human OLFM4 promoter region was performed in LS174T tet-on NICD cells. Cells were stimulated with DOX and TNF-α (50 ng/mL) for 24 h and subjected to ChIP analysis. Immunoprecipitation was performed using either rabbit IgG or anti-NF-κB p65 antibodies. Primer sets were designed to amplify the proximal region of the human OLFM4 promoter, including an area with putative binding sites for RBP-Jk and NF-κB (Site A). Data were normalized to the initial chromatin input. n.s. not significant.
